# Supplementary material for: Simple Donor–π–Acceptor Compounds Exhibiting Aggregation-Induced Emission as Hidden Fingerprints Detecting Agents
Source: Molecules. 2023 Nov 14;28(22):7597. doi: 10.3390/molecules28227597 (PMC10674358; doi:10.3390/molecules28227597)
Supplement: Supplementary file 1 [file molecules-28-07597-s001.zip › molecules-2667569-supplementary.pdf]

Supporting Information

Simple Donor–π–Acceptor Compounds Exhibiting Aggregation-Induced Emission as Hidden Fingerprints Detecting Agents

Patrycja Filipek [1], Hubert Hellwig [2], Agata Szlapa-Kula [1] and Michał Filapek [1,*]

[1] Institute of Chemistry, Faculty of Mathematics, Physics and Chemistry, University of Silesia, Szkolna 9, 40-007 Katowice, Poland
[2] Center for Integrated Technology and Organic Synthesis, Research Unit MolSys, University of Liège, B-4000 Liège, Sart Tilman, Belgium
* Correspondence: michal.filapek@us.edu.pl

Table of Contents

1. **Materials** 2
2. **General methods - measurements** 2
3. **Optical properties** 3
4. **Electrochemical properties** 6
5. **Latent fingerprints** 11
6. **DFT calculations** 13

1. Materials

All chemicals and starting materials were commercially available and were used without further purification. Solvents were distilled as per the standard methods and purged with nitrogen before use. All reactions were carried out under argon atmosphere unless otherwise indicated. Column chromatography was carried out on Merck silica gel. Thin layer chromatography (TLC) was performed on silica gel (Merck TLC Silica Gel 60).

2. General methods - measurements

NMR spectra were recorded with a Bruker Avance 400 MHz instrument by using $CDCl_3$ as a solvent. UV/Vis spectra were recorded with a Hewlett–Packard model 8453 UV/Vis spectrophotometer in the dichloromethane solution. Electrochemical measurements were carried out with an Eco Chemie Autolab PGSTAT128n potentiostat using glassy carbon (with diam. 2 mm) or Indium tin oxide (ITO, with 10 Ω per square) as a working electrode. Platinum coil and silver wire were used as auxiliary and qusireference electrode, respectively. Potentials are referenced to ferrocene (Fc), which was used as the internal standard. Cyclic and differential pulse voltammetry experiments were conducted in a standard one-compartment cell, in CH_2Cl_2 (Carlo Erba, HPLC grade), under argon. Bu_4NPF_6 (Aldrich; 0.2 M, 99%) was used as the supporting electrolyte. UV-Vis spectroelectrochemical measurements were performed on Indium Tin Oxide (ITO) glass working electrode coated with polymers. Polymeric layers were synthesized on an ITO electrode under conditions similar to those of cyclic voltammetry measurement. The quantum theoretical calculations were performed with the use of density functional theory (DFT), with an exchange-correlation hybrid functional CAM-B3LYP and the base 6-311+G. The calculations were carried out with the use of the Gaussian 09 program.

3. Optical properties

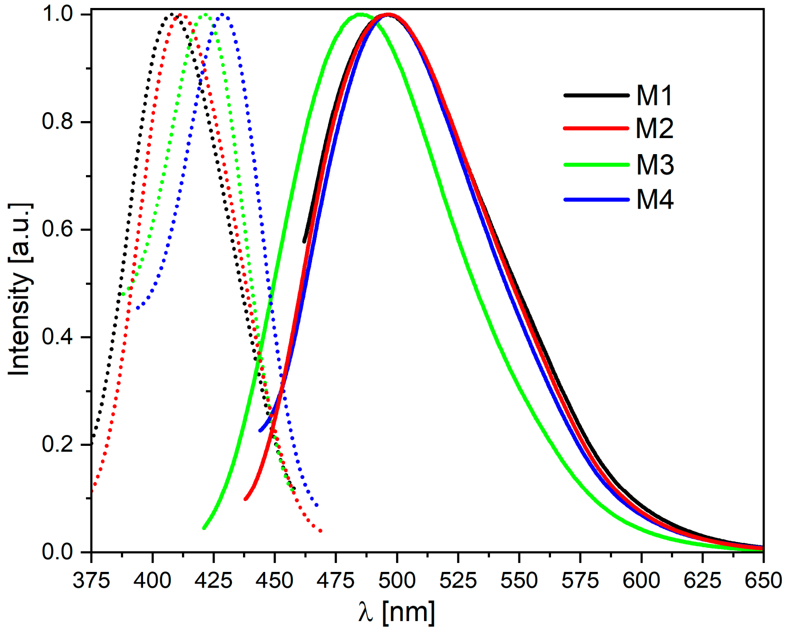

Figure S1. Normalized Emission and Excitation spectra of investigated compounds in THF (c = 10^{-5} M).

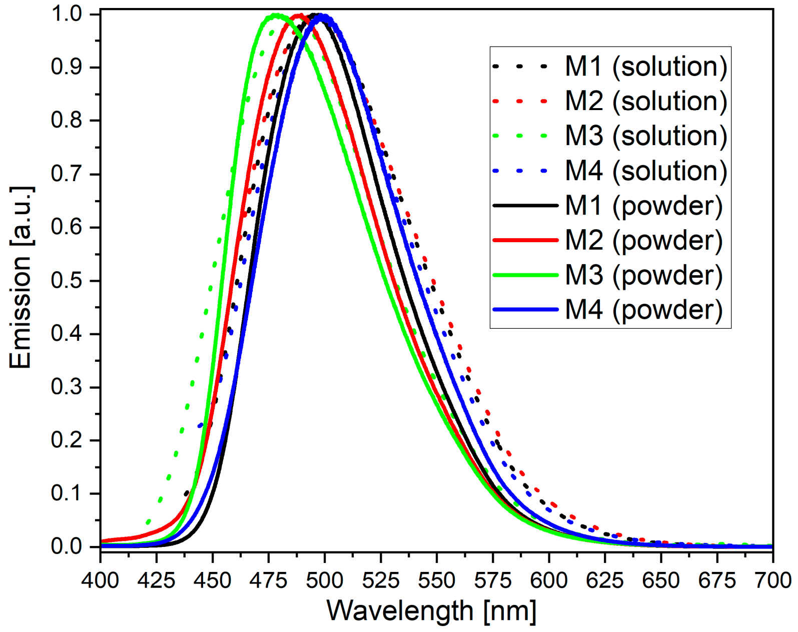

Figure S2. Normalized Emission and Excitation spectra of investigated compounds in THF (c = 10^{-5} M) and as a powder.

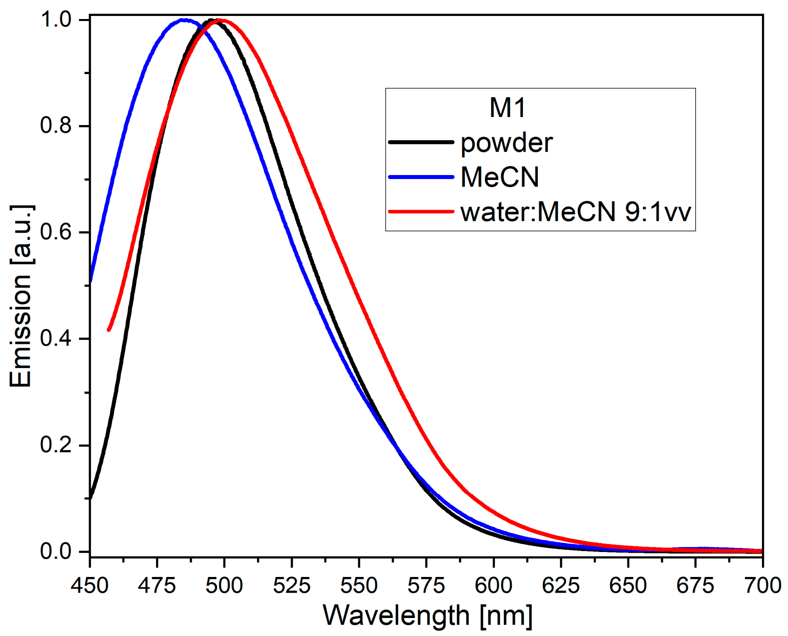

Figure S3. Normalized Emission spectra of M1 in MeCN and water:MeCN mixture (9:1 V/V) (in both cases c = 10^{-5} mol/L) and as a powders.

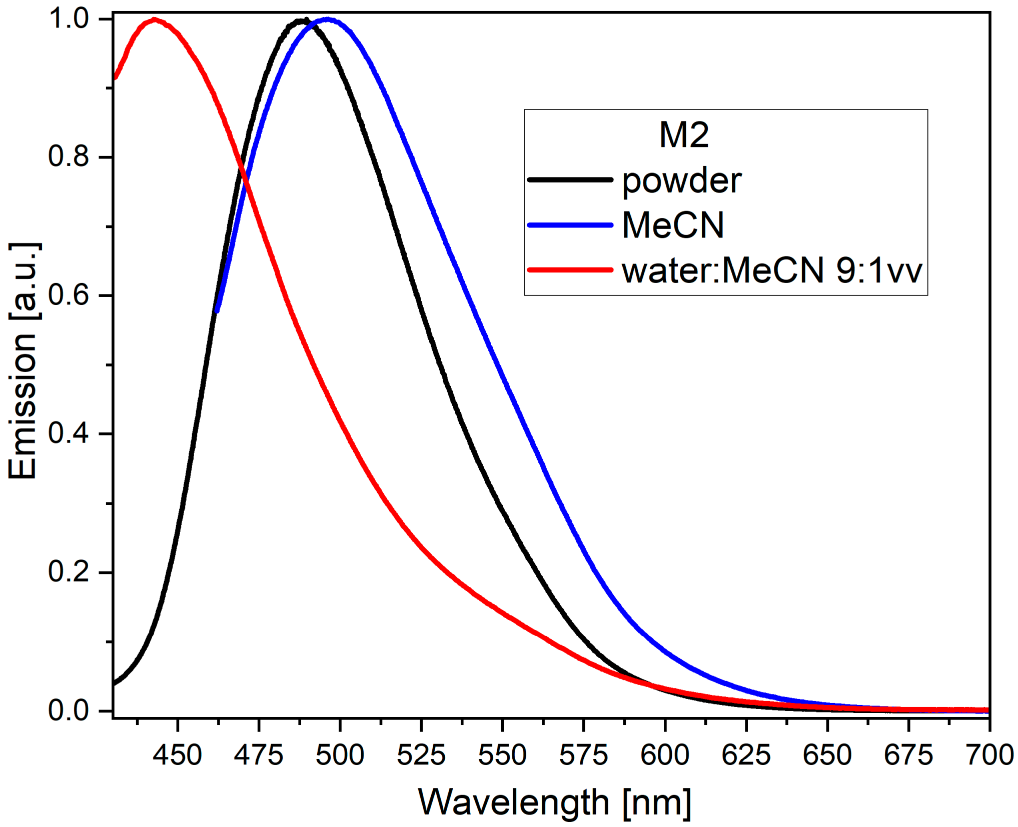

Figure S4. Normalized Emission spectra of M2 in MeCN and water:MeCN mixture (9:1 V/V) (in both cases c = 10^{-5} mol/L) and as a powders.

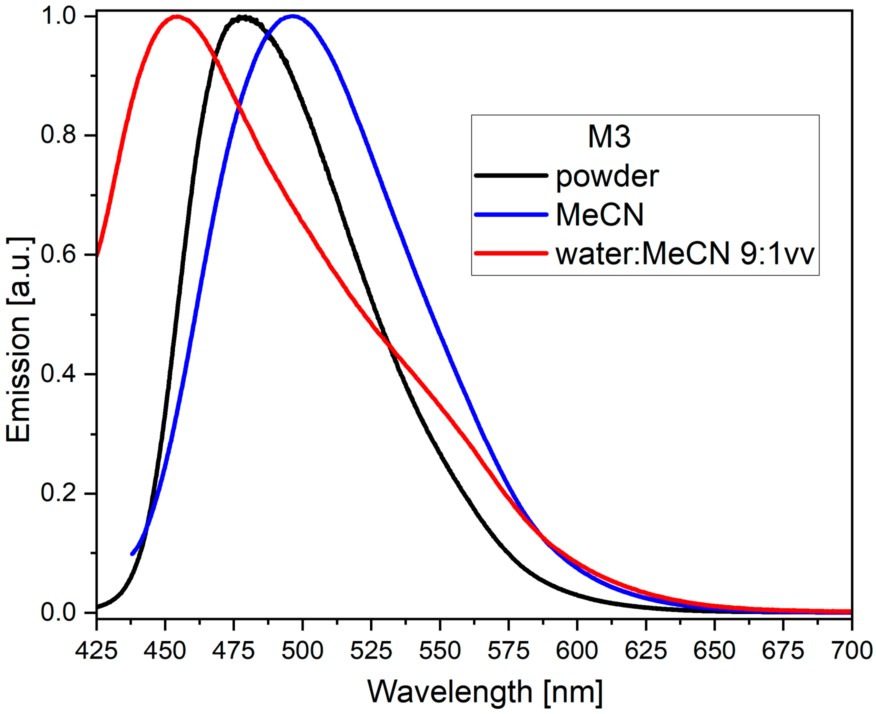

Figure S5. Normalized Emission spectra of M3 in MeCN and water:MeCN mixture (9:1 V/V) (in both cases c = 10^{-5} mol/L) and as a powders.

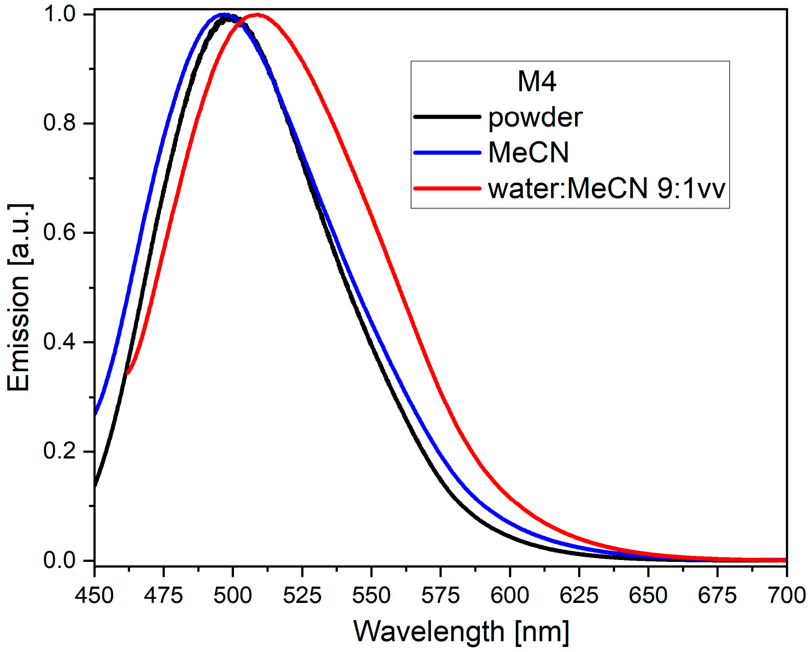

Figure S6. Normalized Emission spectra of M4 in MeCN and water:MeCN mixture (9:1 V/V) (in both cases c = 10^{-5} mol/L) and as a powders.

4. Electrochemical properties

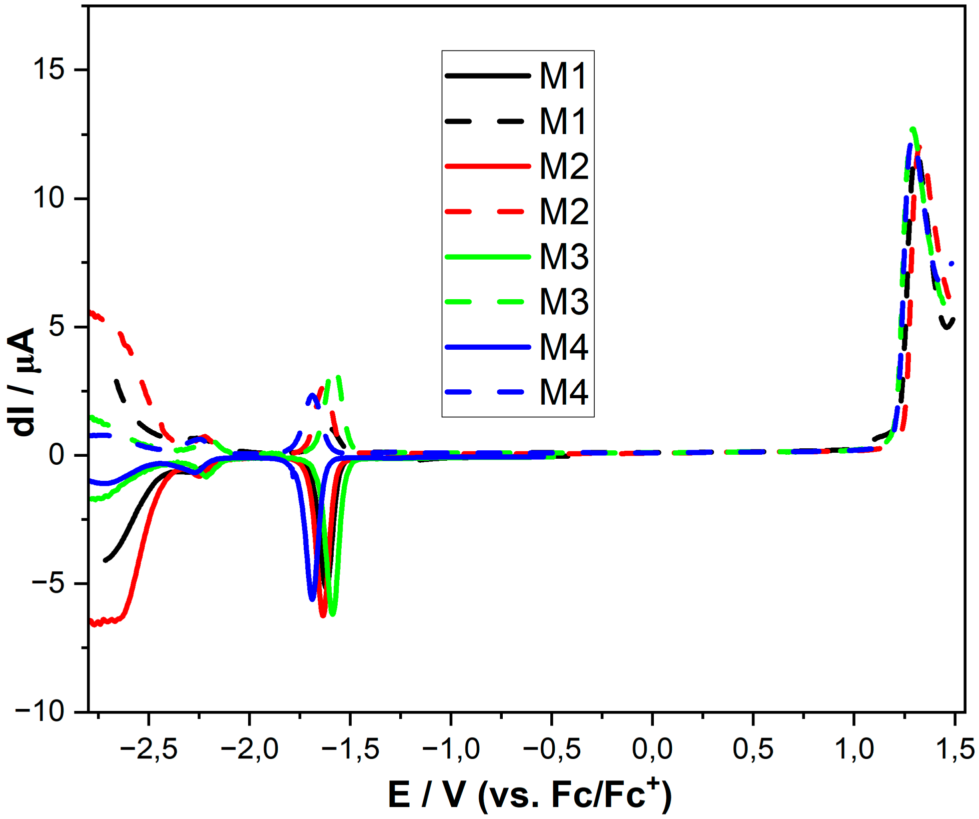

Figure S7. Differential pulse voltammograms of the M1 with different sweep rate. C = 10^{-3} mol/L, 0.1 M Bu_4NPF_6 in CH_2Cl_2.

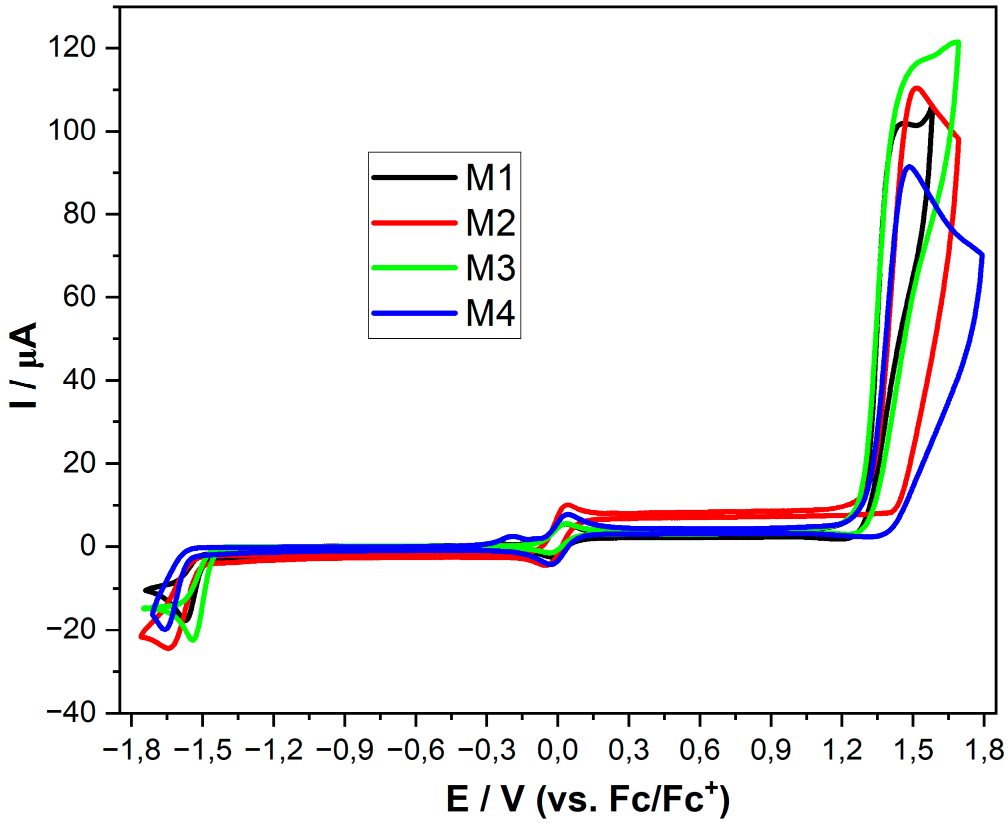

Figure S8. Cyclic voltammograms of the M1-M4 with the addition of the ferrocene (as the internal standard). C = 10^{-3} mol/L, 0.1 M Bu_4NPF_6 in CH_2Cl_2.

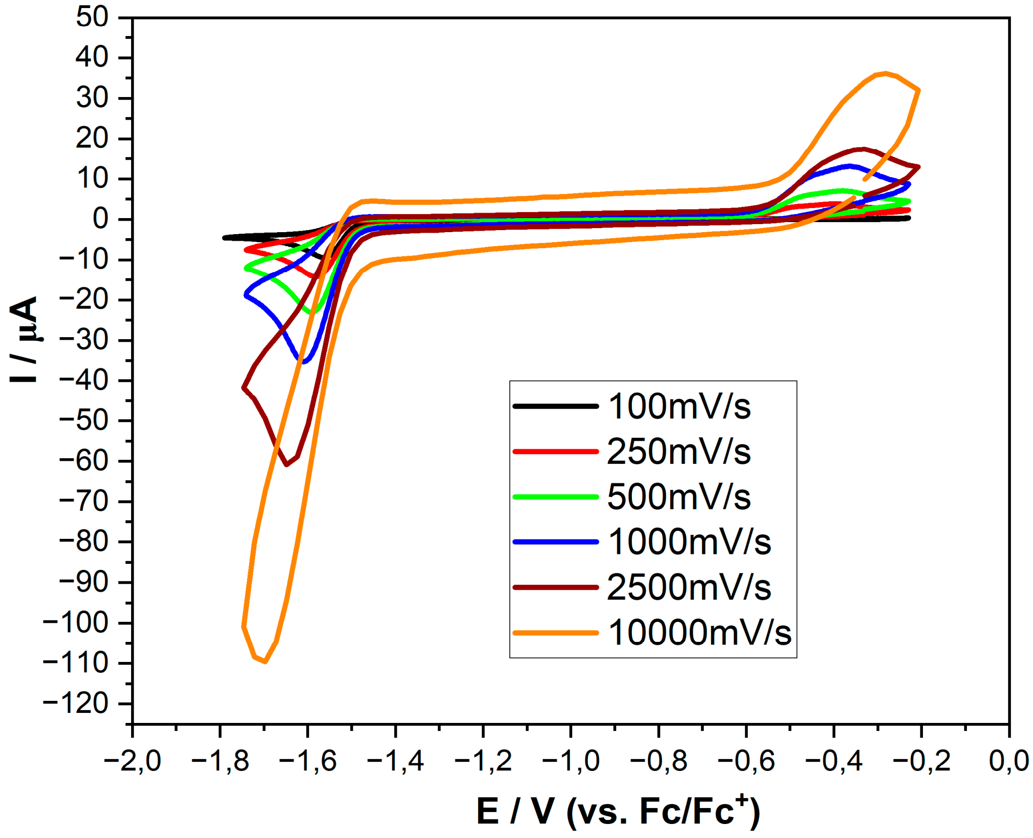

Figure S9. Cyclic voltammograms of the M1 with different sweep rate. C = 10^{-3} mol/L, 0.1 M Bu_4NPF_6 in CH_2Cl_2.

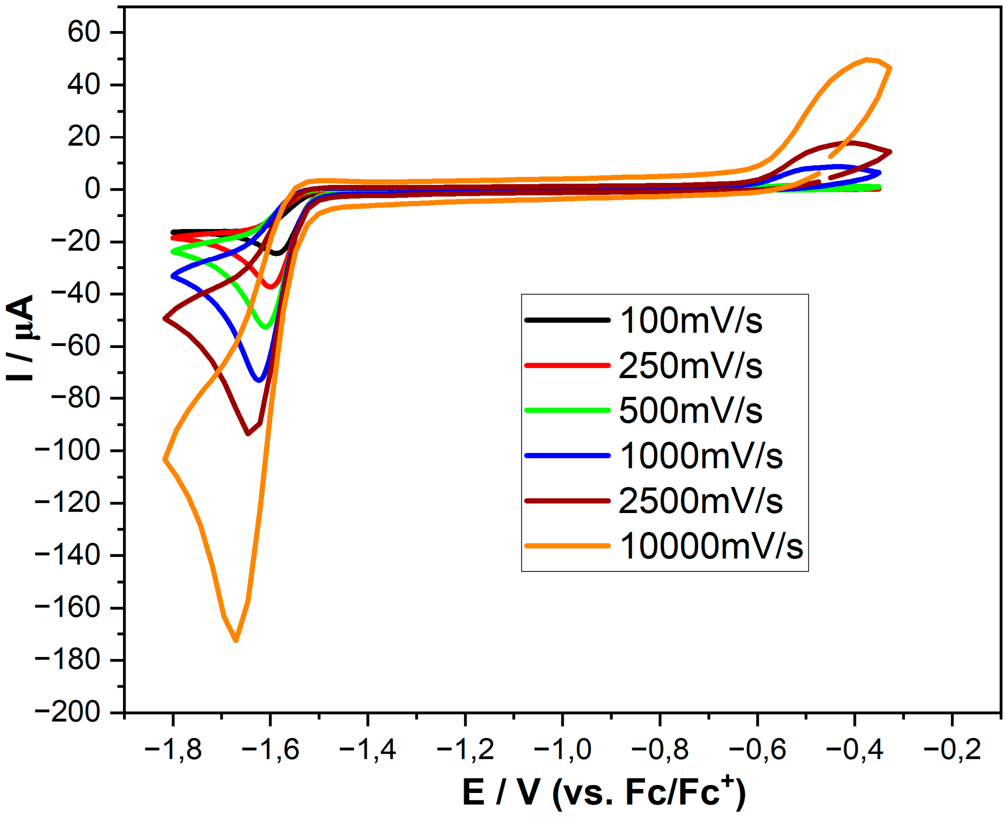

Figure S10. Cyclic voltammograms of the M2 with different sweep rate. C = 10^{-3} mol/L, 0.1 M Bu_4NPF_6 in CH_2Cl_2.

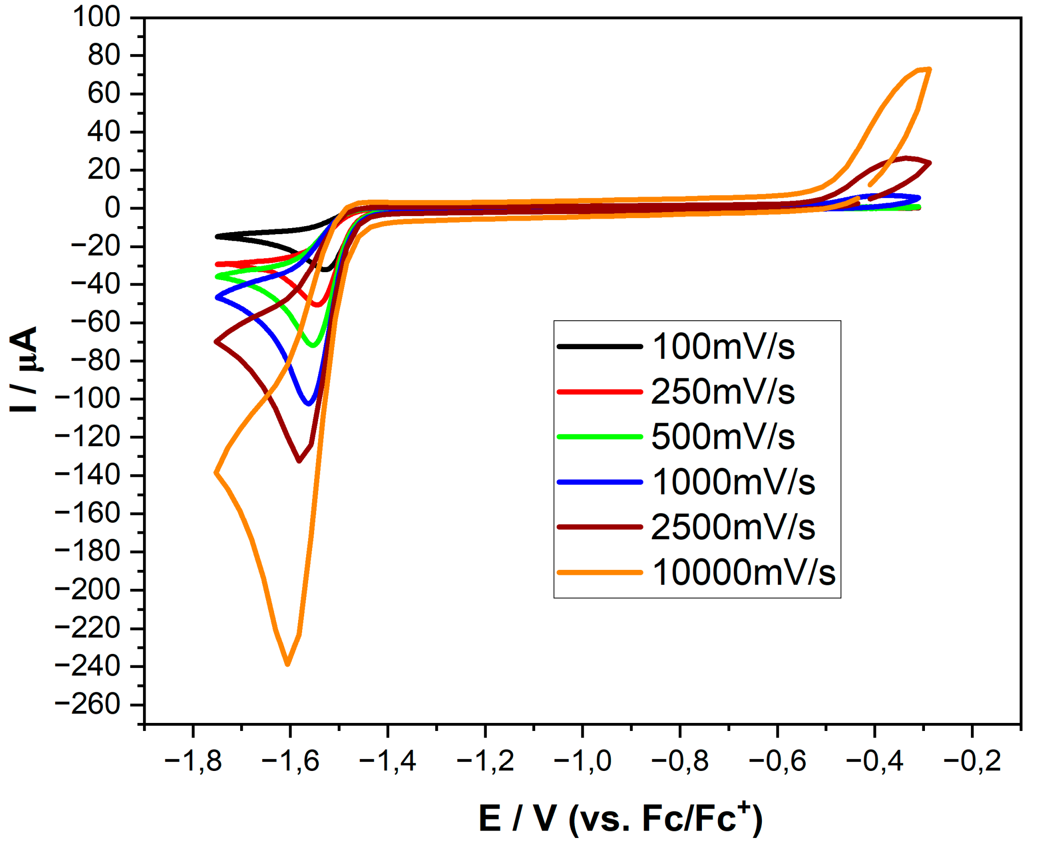

Figure S11. Cyclic voltammograms of the M3 with different sweep rate. C = 10^{-3} mol/L, 0.1 M Bu_4NPF_6 in CH_2Cl_2

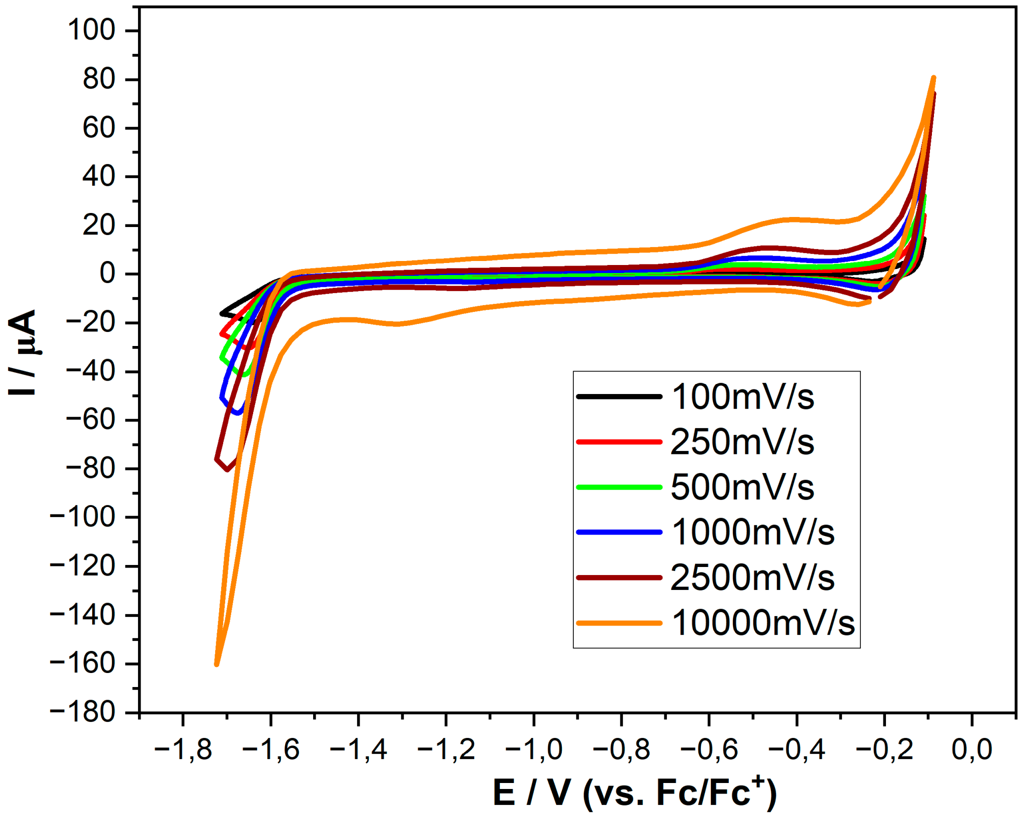

Figure S12. Cyclic voltammograms of the M4 with different sweep rate. C = 10^{-3} mol/L, 0.1 M Bu_4NPF_6 in CH_2Cl_2

5. Latent fingerprints

As already mentioned, in the criminal process, we can distinguish many activities to determine the identity of the perpetrator of a crime. These include DNA testing, osmological tests, as well as polygraph tests. In particular, however, it is worth highlighting that fingerprints are unique biometric features that allow for precise identification of persons. They have several features crucial for conducting a reliable taking of evidence in a criminal trial. Among them, the most important are:

• uniqueness - each person has unique fingerprints. Even in identical twins

fingerprints are different;

• constancy - fingerprints are formed during fetal life and usually remain unchanged throughout life. As the skin ages, only minor changes may occur, but the overall structure of the prints remains the same;

• fingerprint patterns - fingerprints have characteristic fingerprint patterns consisting of loop, delta loop, swirl, and other shapes. These patterns create unique layouts that can be analyzed and compared;
• minutiae - minutiae are characteristic points on fingerprints, such as points of fingerprint intersections or line ends. These points are relevant to the analysis and process fingerprint comparison;
• comparability - fingerprint-based identification systems use algorithms to analyze and compare fingerprint characteristics such as line length fingerprints, the angles between them, and the location of minutiae;
• durability - fingerprints are resistant to many external factors, such as minor injuries, weather conditions, or temporary changes in skin moisture.
• ease of collection - collecting fingerprints is relatively simple and non-invasive. This can be done by using a fingerprint scanner or traditional methods such as inking fingers on paper;
• application - fingerprints are used in areas such as forensics, identity identification, security, access management, border controls, identity documents and much more;
• security - fingerprints as an identification tool are considered secure because these features are difficult to counterfeit or falsify.

Fingerprints play the most crucial role in taking evidence during a criminal trial. They can be a source of information needed to reconstruct the event subject of criminal proceedings. Evidence is crucial to the course of the entire trial, as it is the basis for the indictment. Examination of the body, psychiatric examinations, and fingerprinting constitute legally protected evidence for the proper conduct of the trial.

Table S1 Outline of the scoring schema used to evaluate the developed traces [1].

Points	Detail levels
0	No traces of any mark
1	Poor development; contact proof but no details
2	Limited development; about 1/3 of the details are present but probably cannot be used for identification purposes.
3	Strong development; 1/3 to 2/3 of the details; recognizable finger mark
4	Solid development; full details; recognizable fingerprint

[1] Sears, V. G., Bleay, S. M., Bandey, H. L., Bowman, V. J. A methodology for finger mark research. *Science & Justice*, **2012**, 52, 145-160.

6. DFT Calculations

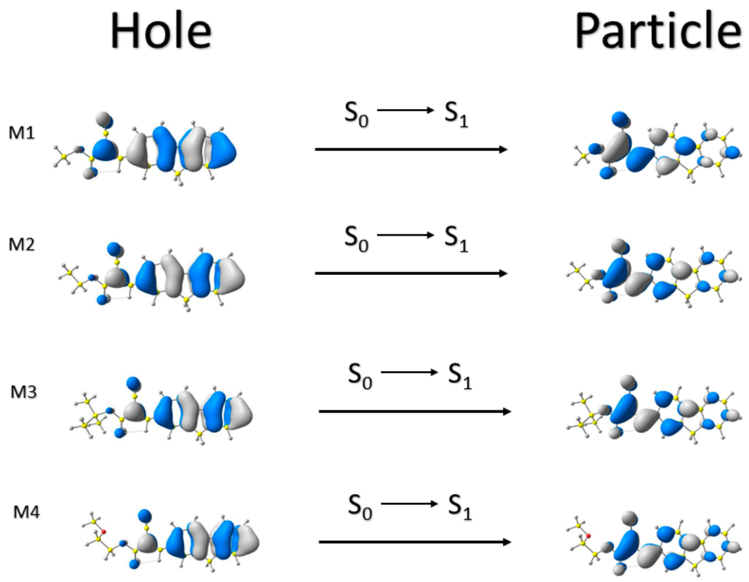

Figure S13. Graphical representation of the S_0 to S_1 transition.
